# Supplementary material for: Both abundant and rare fungi colonizing Fagus sylvatica ectomycorrhizal root-tips shape associated bacterial communities
Source: Commun Biol. 2022 Nov 17;5:1261. doi: 10.1038/s42003-022-04178-y (PMC9672120; doi:10.1038/s42003-022-04178-y)
Supplement: Supplementary file 2 — Supplementary Information [file 42003_2022_4178_MOESM2_ESM.pdf]

Supplementary Information

# Both abundant and rare fungi colonizing *Fagus sylvatica* ectomycorrhizal root-tips shape associated bacterial communities

Marlies Dietrich, Alicia Montesinos-Navarro, Raphael Gabriel, Florian Strasser, Dimitri V. Meier, Werner Mayerhofer, Stefan Gorka, Julia Wiesenbauer, Victoria Martin, Marieluise Weidinger, Andreas Richter, Christina Kaiser, Dagmar Woebken

Supplementary Figure 1 Ternary plots depicting the distribution of all OTUs of (a) fungal and (b) bacterial/archaeal sequences in investigated bulk soil, rhizosphere and mycorrhizal root-tip habitats.

Supplementary Figure 2 Taxonomic composition of (a) fungal and (b) bacterial/archaeal communities associated to bulk soil, rhizosphere and mycorrhizal root-tips habitats.

Supplementary Figure 3 Species richness and diversity of bacterial/archaeal and fungal communities in investigated habitats.

Supplementary Figure 4 Boxplots based on tests of homogeneity of dispersion analysis

Supplementary Figure 5 Identification of abundant and most prevalent fungal OTUs on mycorrhizal root-tip samples.

Supplementary Figure 6 Co-occurring bacterial/archaeal and fungal OTUs across root-tips.

Supplementary Table 1 Number of investigated samples across microenvironments of planted split-root boxes.

Supplementary Table 2 Taxonomic affiliation of 19 fungal OTUs likely involved in morphotype forming on mycorrhizal root-tips.

Supplementary Table 3 Taxonomic affiliation of 4 fungal OTUs and 15 bacterial OTUs co-occurring on all investigated mycorrhizal root-tips.

Supplementary Table 4 Taxonomic affiliation of 40 abundant fungal OTUs present in modules of network “10%”.

Supplementary Table 5 Taxonomic affiliation of 54 abundant fungal OTUs present in modules of network “5%”.

Supplementary Methods 1 Detailed protocols for amplification of 16S rRNA gene.

Supplementary Methods 2 Data analysis and statistics.

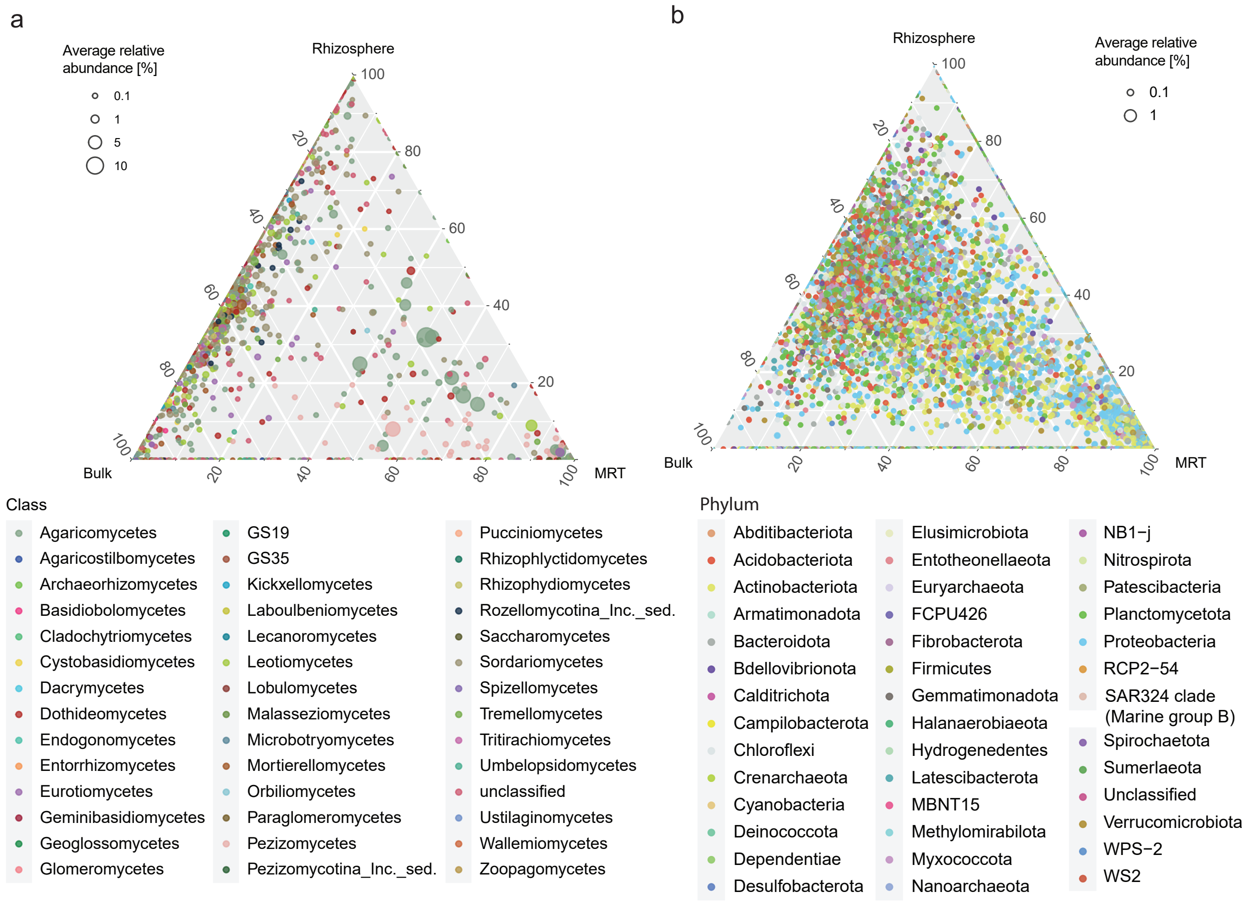

Supplementary Figure 1

Ternary plots depicting the distribution of all OTUs of (a) fungal and (b) bacterial/archaeal sequences in investigating bulk soil, rhizosphere and mycorrhizal root-tip habitats. The dot size corresponds to the average relative abundance of the OTUs. Fungal OTUs are colored by class, bacterial OTUs by phylum. The position of the dots is determined by the contribution of the habitats to the relative abundance of the respective OTU.

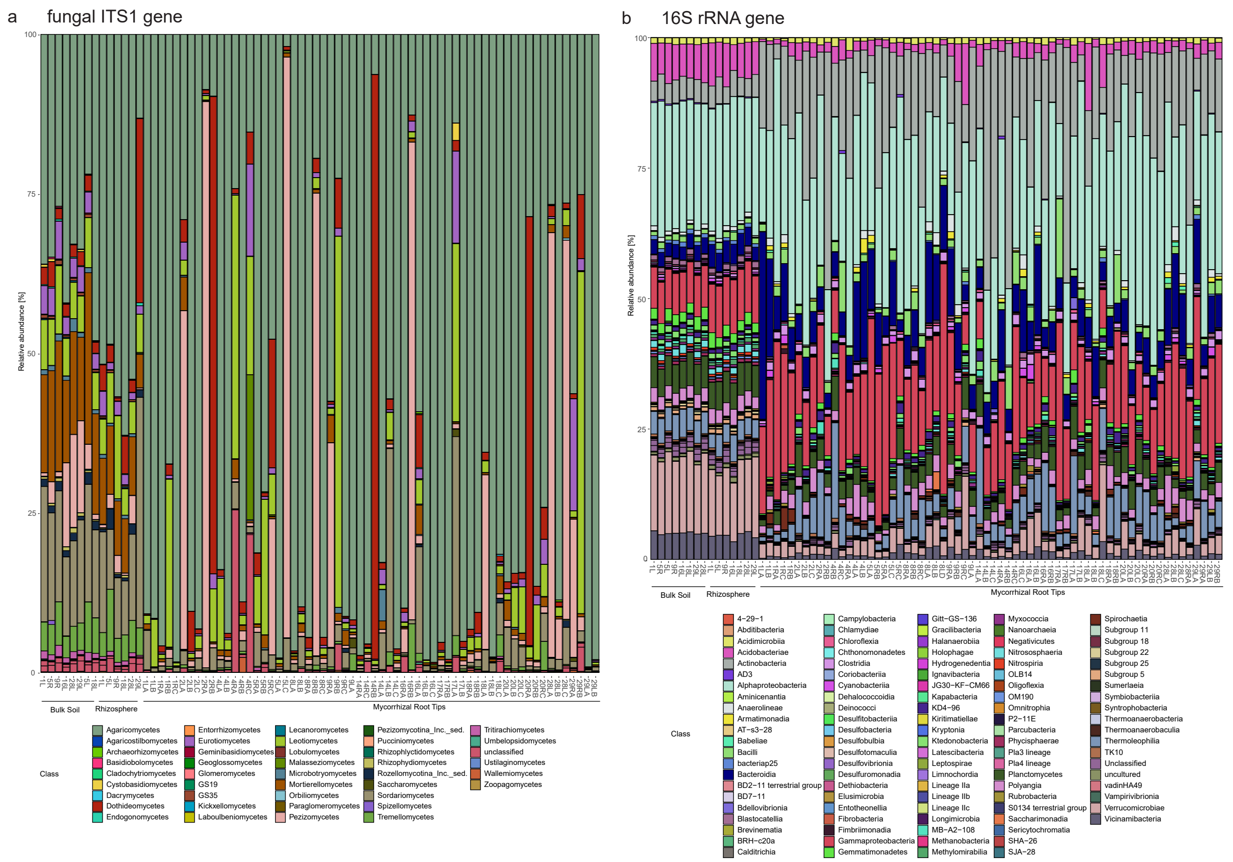

Supplementary Figure 2

Taxonomic composition of (a) fungal and (b) bacterial/archaeal communities associated to bulk soil, rhizosphere and mycorrhizal root tip habitats. Stacked bars depict relative abundances (%) and are colored by class level.

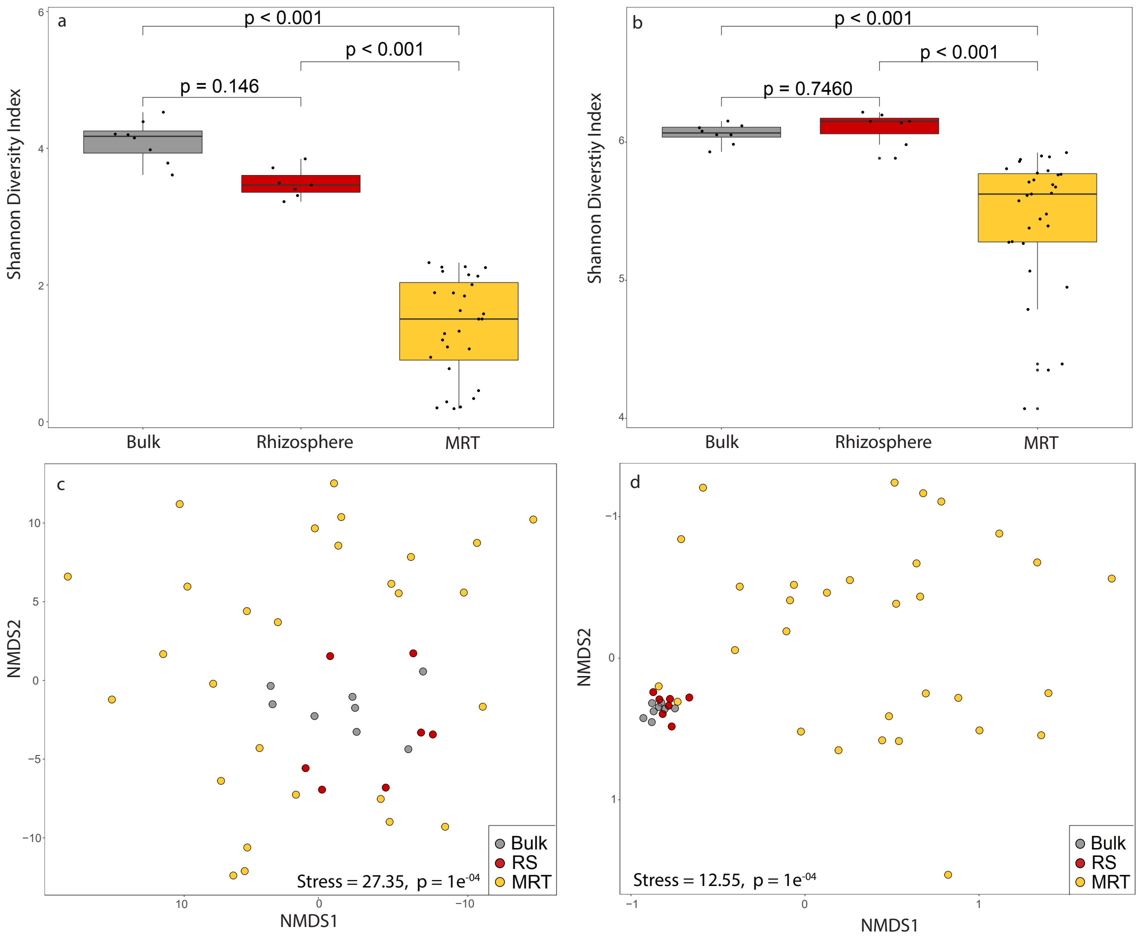

Supplementary Figure 3

Species richness and diversity of bacterial/archaeal and fungal communities in investigated habitats. Species richness of (a) fungal and (b) bacterial/archaeal communities for bulk soil (Bulk, grey), rhizosphere soil (RS, red) and mycorrhizal root-tips (MRT, yellow) based on Shannon diversity index. P-values based on (a) ANOVA - TukeyHSD test and (b) Kruskal Wallis - Dunn test indicated. Lower and upper hinges indicate the first and third quartiles. Two-dimensional, non-metric dimensional scaling (NMDS) ordination plots of (c) fungal and (d) bacterial/archaeal communities illustrate beta-diversity of obtained samples from bulk, rhizosphere and mycorrhizal root-tip environment based on Bray-Curtis metric (Permanova test). Exact sample sizes, bulk soil (n=8 biologically independent samples), rhizosphere soil (n=7 biologically independent samples), mycorrhizal root tip (n=31 biologically independent samples).

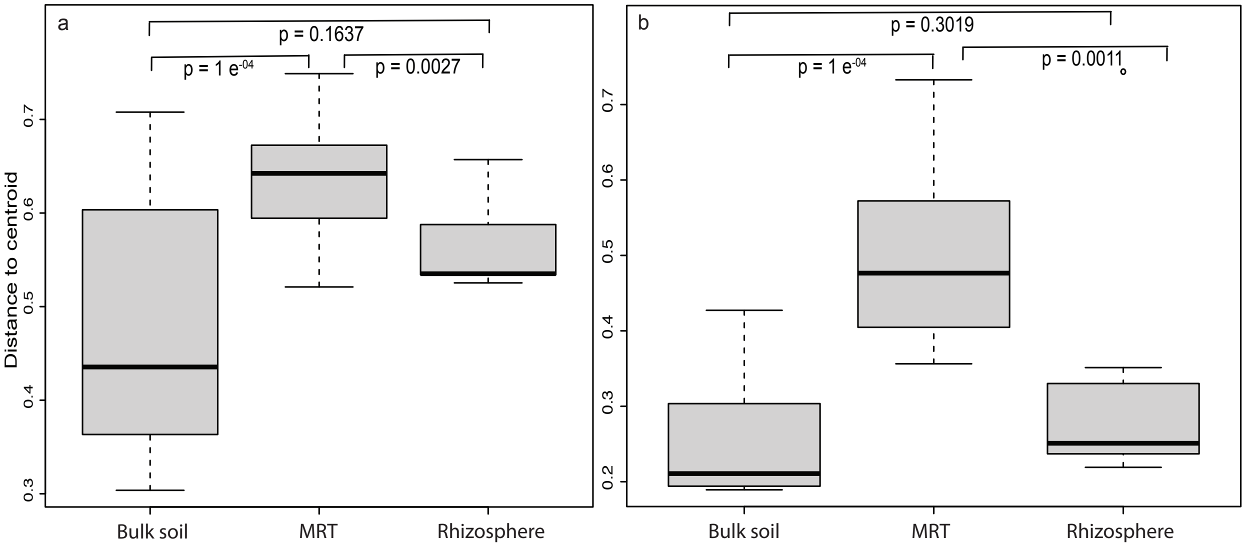

Supplementary Figure 4

Boxplots based on tests of homogeneity of dispersion analysis represent main distances from group centroids for (a) fungal ITS community data and (b)16S community data for bulk soil, rhizosphere and mycorrhizal root-tips (MRT). P-values of pairwise comparisons of permutation tests for homogeneity of multivariate dispersions (Permidist run with 9999 permutations) are indicated. Lower and upper hinges indicate the first and third quartiles, for exact sample size see Supplementary Table 1. Exact sample sizes, bulk soil (n=8 biologically independent samples), rhizosphere soil (n=7 biologically independent samples), mycorrhizal root tip (n=31 biologically independent samples).

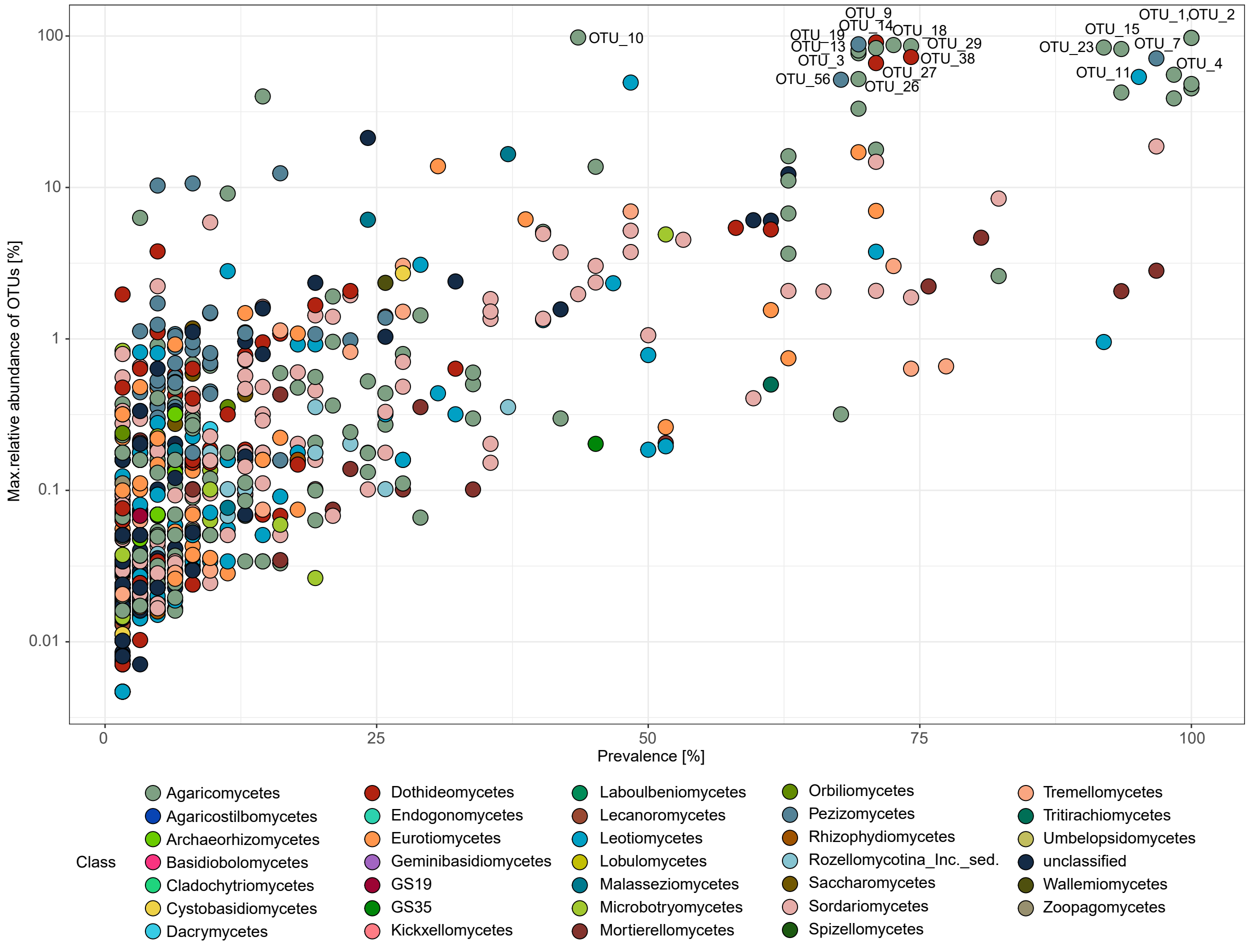

Supplementary Figure 5

Identification of abundant and most prevalent fungal OTUs on mycorrhizal root-tip samples. Scatter plot depicts the highest relative abundance [%] of each fungal OTU among all root tips and the prevalence [%] across all 62 investigated samples. The 19 labeled OTUs had a relative abundance of more than 50% on individual root-tips and are thus likely members of the morphotype forming fungi.

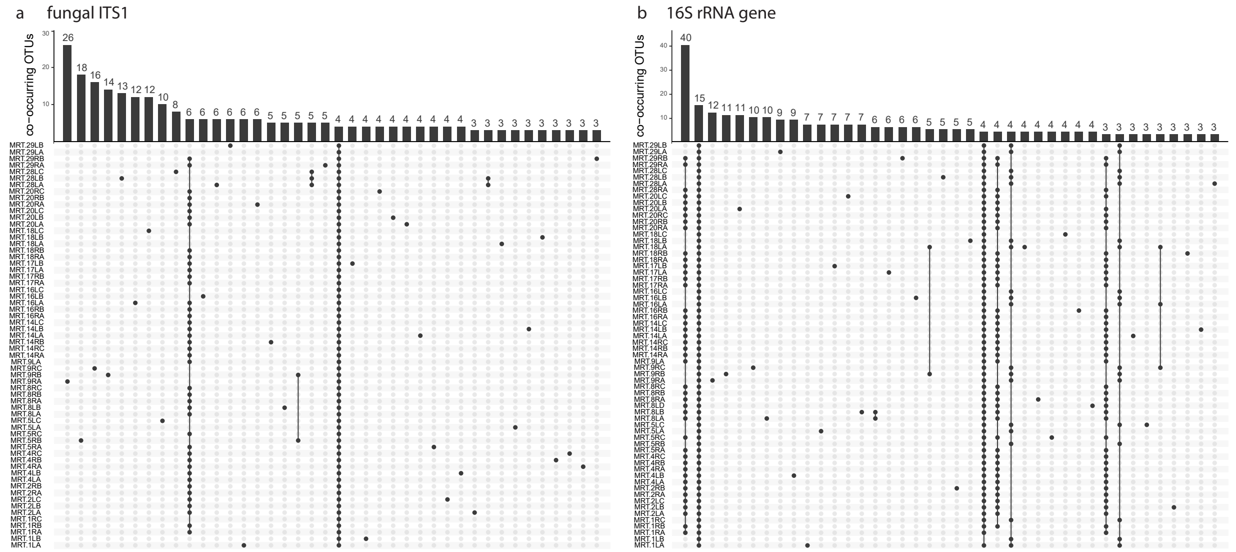

Supplementary Figure 6

Co-occurring bacterial/archaeal and fungal OTUs across root-tips. Matrix layout of Upset plot depicting 40 quantitative intersections (co-occurring OTUs) of (A) fungal OTUs and (B) bacterial/archaeal OTUs in 62 mycorrhizal root-tip samples. Interactions are sorted by size (=number of OTUs in top bars). Black dots in the matrix indicate the presence of the interaction in the respective mycorrhizal root-tip sample listed on the left.

Supplementary Table 1

Number of investigated samples across microenvironments of planted split-root boxes.

Box	Bulk Soil	Rhizosphere	Mycorrhizal Root Tips	additional MRTs Box	MRT
1	1	1	5	2	5
5	2	1	5	4	5
9	1	1	4	8	5
16	1	1	5	14	6
18	1	1	5	17	4
28	1	1	3	20	6
29	1	1	4		
SUM [n=]	8	7	31	SUM [n=]	31

Supplementary Table 2

Taxonomic affiliation of 19 fungal OTUs liklely involved in morphotype forming on mycorrhizal root-tips.

OTU	Kingdom	Phylum	Class	Order	Family	Genus	Species	ECM
OTU_1	Fungi	Basidiomycota	Agaricomycetes	Thelephorales	Thelephoraceae	unclassified	unclassified	ECM
OTU_2	Fungi	Basidiomycota	Agaricomycetes	Agaricales	Inocybaceae	Inocybe	Inocybe margaritispora	ECM
OTU_3	Fungi	Basidiomycota	Agaricomycetes	Thelephorales	Thelephoraceae	Tomentella	Tomentella punicea	ECM
OTU_4	Fungi	Basidiomycota	Agaricomycetes	Thelephorales	Thelephoraceae	Tomentella	Tomentella papuae	ECM
OTU_7	Fungi	Ascomycota	Pezizomycetes	Pezizales	Pezizaceae	Peziza	Peziza ostracoderma	Other
OTU_9	Fungi	Ascomycota	Dothideomycetes	Mytilinidales	Gloniaceae	Cenococcum	Cenococcum geophilum	ECM
OTU_10	Fungi	Basidiomycota	Agaricomycetes	Sebacinales	Sebacinaceae	Helvellosebacina	Helvellosebacina	Other
OTU_11	Fungi	Ascomycota	Leotiomycetes	Helotiales	Hyaloscyphaceae	unclassified	unclassified	Other
OTU_13	Fungi	Basidiomycota	Agaricomycetes	Thelephorales	Thelephoraceae	Tomentella	Tomentella	ECM
OTU_14	Fungi	Basidiomycota	Agaricomycetes	Thelephorales	Thelephoraceae	Tomentella	Tomentella punicea	ECM
OTU_15	Fungi	Basidiomycota	Agaricomycetes	Sebacinales	Serendipitaceae	Serendipita	Serendipita	Other
OTU_18	Fungi	Basidiomycota	Agaricomycetes	Russulales	Russulaceae	Lactarius	Lactarius subdulcis	ECM
OTU_19	Fungi	Ascomycota	Pezizomycetes	Pezizales	Tuberaceae	Tuber	Tuber	ECM
OTU_23	Fungi	Basidiomycota	Agaricomycetes	Agaricales	Hydnangiaceae	Laccaria	Laccaria laccata	ECM
OTU_26	Fungi	Basidiomycota	Agaricomycetes	Sebacinales	Sebacinaceae	Sebacina	Sebacina	ECM
OTU_27	Fungi	Ascomycota	Dothideomycetes	Pleosporales	Didymellaceae	Epicoccum	Epicoccum dendrobii	ECM
OTU_29	Fungi	Basidiomycota	Agaricomycetes	Thelephorales	Thelephoraceae	Thelephora	Thelephora terrestris	ECM
OTU_38	Fungi	Ascomycota	Dothideomycetes	Mytilinidales	Gloniaceae	Cenococcum	Cenococcum geophilum	ECM
OTU_56	Fungi	Ascomycota	Pezizomycetes	Pezizales	Helvellaceae	Helvella	Helvella elastica	ECM

Supplementary Table 3
Taxonomic affiliation of 4 fungal OTUs and 15 bacterial OTUs co-occurring on all investigated mycorrhizal root-tips.

OTU	Phylum	Class	Order	Family	Genus
Shared fungal OTUs					
OTU_2	Basidiomycota	Agaricomycetes	Agaricales	Inocybaceae	Inocybe
OTU_17	Basidiomycota	Agaricomycetes	Agaricales	unclassified	unclassified
OTU_1	Basidiomycota	Agaricomycetes	Thelephorales	Thelephoraceae	unclassified
OTU_12	Basidiomycota	Agaricomycetes	Thelephorales	Thelephoraceae	Tomentella
Shared bacterial OTUs					
OTU_200	Firmicutes	Bacilli	Bacillales	Bacillaceae	Bacillus
OTU_9	Proteobacteria	Gammaproteobacteria	Burkholderiales	Comamonadaceae	Unclassified
OTU_114	Proteobacteria	Gammaproteobacteria	Burkholderiales	Comamonadaceae	Unclassified
OTU_12	Verrucomicrobiota	Verrucomicrobiae	Chthoniobacterales	Chthoniobacteraceae	Candidatus Udaeobacter
OTU_80	Actinobacteriota	Actinobacteria	Corynebacteriales	Mycobacteriaceae	Mycobacterium
OTU_77	Actinobacteriota	Actinobacteria	Frankiales	Acidothermaceae	Acidothermus
OTU_5	Proteobacteria	Alphaproteobacteria	Rhizobiales	Xanthobacteraceae	Bradyrhizobium
OTU_10	Proteobacteria	Alphaproteobacteria	Rhizobiales	Rhizobiaceae	Rhizobium
OTU_11	Proteobacteria	Alphaproteobacteria	Rhizobiales	Xanthobacteraceae	uncultured
OTU_16	Proteobacteria	Alphaproteobacteria	Rhizobiales	Methyloligellaceae	uncultured
OTU_17	Proteobacteria	Alphaproteobacteria	Rhizobiales	Devosiaceae	Unclassified
OTU_31	Proteobacteria	Alphaproteobacteria	Rhizobiales	Rhizobiaceae	Unclassified
OTU_36	Proteobacteria	Alphaproteobacteria	Rhizobiales	Xanthobacteraceae	Unclassified
OTU_42	Proteobacteria	Alphaproteobacteria	Rhizobiales	Xanthobacteraceae	Bradyrhizobium
OTU_48	Proteobacteria	Alphaproteobacteria	Rhizobiales	Rhizobiaceae	Mesorhizobium

Supplementary Table 4

Taxonomic affiliation of 40 abundant fungal OTUs present in modules of network “10%”.

OTU	Kingdom	Phylum	Class	Order	Family	Genus	module
OTU_38	Fungi	Ascomycota	Dothideomycetes	Mytilinidales	Gloniaceae	Cenococcum	3
OTU_9	Fungi	Ascomycota	Dothideomycetes	Mytilinidales	Gloniaceae	Cenococcum	4
OTU_56	Fungi	Ascomycota	Pezizomycetes	Pezizales	Helvellaceae	Helvella	2
OTU_117	Fungi	Ascomycota	Pezizomycetes	Pezizales	Discinaceae	Hydnotrya	1
OTU_7	Fungi	Ascomycota	Pezizomycetes	Pezizales	Pezizaceae	Peziza	1
OTU_19	Fungi	Ascomycota	Pezizomycetes	Pezizales	Tuberaceae	Tuber	4
OTU_390	Fungi	Ascomycota	Pezizomycetes	Pezizales	Tuberaceae	Tuber	1
OTU_10	Fungi	Basidiomycota	Agaricomycetes	Sebacinales	Sebacinaceae	Helvellosebacina	1
OTU_2	Fungi	Basidiomycota	Agaricomycetes	Agaricales	Inocybaceae	Inocybe	1
OTU_23	Fungi	Basidiomycota	Agaricomycetes	Agaricales	Hydnangiaceae	Laccaria	2
OTU_25	Fungi	Basidiomycota	Agaricomycetes	Agaricales	Hydnangiaceae	Laccaria	1
OTU_18	Fungi	Basidiomycota	Agaricomycetes	Russulales	Russulaceae	Lactarius	2
OTU_65	Fungi	Basidiomycota	Agaricomycetes	Boletales	Melanogastraceae	Melanogaster	4
OTU_70	Fungi	Basidiomycota	Agaricomycetes	Agaricales	Tricholomataceae	Mycenella	2
OTU_121	Fungi	Basidiomycota	Agaricomycetes	Sebacinales	Sebacinaceae	Sebacina	2
OTU_26	Fungi	Basidiomycota	Agaricomycetes	Sebacinales	Sebacinaceae	Sebacina	2
OTU_15	Fungi	Basidiomycota	Agaricomycetes	Sebacinales	Serendipitaceae	Serendipita	4
OTU_29	Fungi	Basidiomycota	Agaricomycetes	Thelephorales	Thelephoraceae	Thelephora	4
OTU_12	Fungi	Basidiomycota	Agaricomycetes	Thelephorales	Thelephoraceae	Tomentella	4
OTU_13	Fungi	Basidiomycota	Agaricomycetes	Thelephorales	Thelephoraceae	Tomentella	4
OTU_14	Fungi	Basidiomycota	Agaricomycetes	Thelephorales	Thelephoraceae	Tomentella	3
OTU_3	Fungi	Basidiomycota	Agaricomycetes	Thelephorales	Thelephoraceae	Tomentella	4
OTU_32	Fungi	Basidiomycota	Agaricomycetes	Thelephorales	Thelephoraceae	Tomentella	4
OTU_4	Fungi	Basidiomycota	Agaricomycetes	Thelephorales	Thelephoraceae	Tomentella	4
OTU_73	Fungi	Basidiomycota	Agaricomycetes	Thelephorales	Thelephoraceae	Tomentella	1
OTU_1	Fungi	Basidiomycota	Agaricomycetes	Thelephorales	Thelephoraceae	unclassified	1
OTU_47	Fungi	Ascomycota	Eurotiomycetes	Eurotiales	Aspergillaceae	Aspergillus	4
OTU_27	Fungi	Ascomycota	Dothideomycetes	Pleosporales	Didymellaceae	Epicoccum	4
OTU_182	Fungi	Ascomycota	Leotiomycetes	Helotiales	Hyaloscyphaceae	Hyaloscypha	4
OTU_235	Fungi	Ascomycota	Eurotiomycetes	Eurotiales	Trichocomaceae	Talaromyces	4
OTU_6	Fungi	Ascomycota	Sordariomycetes	Hypocreales	Hypocreaceae	Trichoderma	4
OTU_11	Fungi	Ascomycota	Leotiomycetes	Helotiales	Hyaloscyphaceae	unclassified	4
OTU_324	Fungi	Ascomycota	Pezizomycetes	Pezizales	Tuberaceae	unclassified	1
OTU_81	Fungi	Ascomycota	Sordariomycetes	Chaetosphaeriales	Chaetosphaeriaceae	unclassified	4
OTU_58	Fungi	Ascomycota	unclassified	unclassified	unclassified	unclassified	4
OTU_80	Fungi	Basidiomycota	Agaricomycetes	Agaricales	Tricholomataceae	Hydropus	1
OTU_245	Fungi	Basidiomycota	Malasseziomycetes	Malasseziales	Malasseziaceae	Malassezia	4
OTU_17	Fungi	Basidiomycota	Agaricomycetes	Agaricales	unclassified	unclassified	3
OTU_20	Fungi	Basidiomycota	Agaricomycetes	Agaricales	Hebelomataceae	unclassified	3
OTU_209	Fungi	unclassified	unclassified	unclassified	unclassified	unclassified	4

Supplementary Table 5

Taxonomic affiliation of 54 abundant fungal OTUs present in modules of network "5%".

OTU	Kingdom	Phylum	Class	Order	Family	Genus	Module
OTU_47	Fungi	Ascomycota	Eurotiomycetes	Eurotiales	Aspergillaceae	Aspergillus	6
OTU_123	Fungi	Ascomycota	Dothideomycetes	Mytilinidales	Gloniaceae	Cenococcum	6
OTU_38	Fungi	Ascomycota	Dothideomycetes	Mytilinidales	Gloniaceae	Cenococcum	3
OTU_9	Fungi	Ascomycota	Dothideomycetes	Mytilinidales	Gloniaceae	Cenococcum	5
OTU_157	Fungi	Ascomycota	Eurotiomycetes	Chaetothyriales	Herpotrichiellaceae	Cladophialophora	4
OTU_86	Fungi	Ascomycota	Eurotiomycetes	Chaetothyriales	Herpotrichiellaceae	Cladophialophora	4
OTU_27	Fungi	Ascomycota	Dothideomycetes	Pleosporales	Didymellaceae	Epicoccum	4
OTU_56	Fungi	Ascomycota	Pezizomycetes	Pezizales	Helvellaceae	Helvella	3
OTU_182	Fungi	Ascomycota	Leotiomycetes	Helotiales	Hyaloscyphaceae	Hyaloscypha	5
OTU_117	Fungi	Ascomycota	Pezizomycetes	Pezizales	Discinaceae	Hydnotrya	2
OTU_45	Fungi	Ascomycota	Sordariomycetes	Hypocreales	Clavicipitaceae	Metarhizium	5
OTU_107	Fungi	Ascomycota	Dothideomycetes	Capnodiales	Mycosphaerellaceae	Mycosphaerella	3
OTU_7	Fungi	Ascomycota	Pezizomycetes	Pezizales	Pezizaceae	Peziza	2
OTU_235	Fungi	Ascomycota	Eurotiomycetes	Eurotiales	Trichocomaceae	Talaromyces	4
OTU_105	Fungi	Ascomycota	Sordariomycetes	Sordariales	Chaetomiaceae	Trichocladium	4
OTU_6	Fungi	Ascomycota	Sordariomycetes	Hypocreales	Hypocreaceae	Trichoderma	5
OTU_19	Fungi	Ascomycota	Pezizomycetes	Pezizales	Tuberaceae	Tuber	6
OTU_390	Fungi	Ascomycota	Pezizomycetes	Pezizales	Tuberaceae	Tuber	2
OTU_11	Fungi	Ascomycota	Leotiomycetes	Helotiales	Hyaloscyphaceae	unclassified	4
OTU_324	Fungi	Ascomycota	Pezizomycetes	Pezizales	Tuberaceae	unclassified	2
OTU_58	Fungi	Ascomycota	unclassified	unclassified	unclassified	unclassified	4
OTU_77	Fungi	Ascomycota	unclassified	unclassified	unclassified	unclassified	4
OTU_81	Fungi	Ascomycota	Sordariomycetes	Chaetosphaeriales	Chaetosphaeriaceae	unclassified	4
OTU_812	Fungi	Ascomycota	Sordariomycetes	Hypocreales	unclassified	unclassified	2
OTU_91	Fungi	Ascomycota	unclassified	unclassified	unclassified	unclassified	4
OTU_131	Fungi	Basidiomycota	Agaricomycetes	Agaricales	Tricholomataceae	Delicatula	2
OTU_492	Fungi	Basidiomycota	Agaricomycetes	Agaricales	Entolomataceae	Entoloma	2
OTU_10	Fungi	Basidiomycota	Agaricomycetes	Sebacinales	Sebacinaceae	Helvellosebacina	2
OTU_80	Fungi	Basidiomycota	Agaricomycetes	Agaricales	Tricholomataceae	Hydropus	2
OTU_2	Fungi	Basidiomycota	Agaricomycetes	Agaricales	Inocybaceae	Inocybe	2
OTU_23	Fungi	Basidiomycota	Agaricomycetes	Agaricales	Hydnangiaceae	Laccaria	6
OTU_25	Fungi	Basidiomycota	Agaricomycetes	Agaricales	Hydnangiaceae	Laccaria	2
OTU_18	Fungi	Basidiomycota	Agaricomycetes	Russulales	Russulaceae	Lactarius	6
OTU_245	Fungi	Basidiomycota	Malasseziomycetes	Malasseziales	Malasseziaceae	Malassezia	4
OTU_498	Fungi	Basidiomycota	Malasseziomycetes	Malasseziales	Malasseziaceae	Malassezia	4
OTU_65	Fungi	Basidiomycota	Agaricomycetes	Boletales	Melanogastraceae	Melanogaster	4
OTU_70	Fungi	Basidiomycota	Agaricomycetes	Agaricales	Tricholomataceae	Mycenella	5
OTU_130	Fungi	Basidiomycota	Tremellomycetes	Filobasidiales	Filobasidiaceae	Naganishia	6
OTU_121	Fungi	Basidiomycota	Agaricomycetes	Sebacinales	Sebacinaceae	Sebacina	5
OTU_26	Fungi	Basidiomycota	Agaricomycetes	Sebacinales	Sebacinaceae	Sebacina	5
OTU_15	Fungi	Basidiomycota	Agaricomycetes	Sebacinales	Serendipitaceae	Serendipita	4
OTU_83	Fungi	Basidiomycota	Agaricomycetes	Sebacinales	Serendipitaceae	Serendipita	4
OTU_29	Fungi	Basidiomycota	Agaricomycetes	Thelephorales	Thelephoraceae	Thelephora	5
OTU_12	Fungi	Basidiomycota	Agaricomycetes	Thelephorales	Thelephoraceae	Tomentella	1
OTU_13	Fungi	Basidiomycota	Agaricomycetes	Thelephorales	Thelephoraceae	Tomentella	5
OTU_14	Fungi	Basidiomycota	Agaricomycetes	Thelephorales	Thelephoraceae	Tomentella	6
OTU_3	Fungi	Basidiomycota	Agaricomycetes	Thelephorales	Thelephoraceae	Tomentella	4
OTU_32	Fungi	Basidiomycota	Agaricomycetes	Thelephorales	Thelephoraceae	Tomentella	6
OTU_4	Fungi	Basidiomycota	Agaricomycetes	Thelephorales	Thelephoraceae	Tomentella	1
OTU_73	Fungi	Basidiomycota	Agaricomycetes	Thelephorales	Thelephoraceae	Tomentella	2
OTU_1	Fungi	Basidiomycota	Agaricomycetes	Thelephorales	Thelephoraceae	unclassified	2
OTU_17	Fungi	Basidiomycota	Agaricomycetes	Agaricales	unclassified	unclassified	3
OTU_20	Fungi	Basidiomycota	Agaricomycetes	Agaricales	Hebelomataceae	unclassified	3
OTU_209	Fungi	unclassified	unclassified	unclassified	unclassified	unclassified	4

Supplementary Methods 1

The 16S rRNA gene was amplified via polymerase chain reaction (PCR) using a multiplexed barcoding amplicon sequencing approach. During the first step, the 16S rRNA was amplified in triplicates using 22 cycles. Each 20 µl PCR mix consisted of 12.72/13.72 µl nuclease free water, 2 µl 10x DreamTaq Green Buffer, 2 µl 2 mM dNTP mix, 0.08 µl BSA (0.08 µg $µl^{-1}$), 0.2 µl of 1.25U DreamTaq Green DNA Polymerase, 0.5 µl 10 µM of each primer, and 1 or 2 µl DNA template (ca. 10 ng per reaction). 2 µl DNA template were used for rhizoplane samples and mycorrhizal root tips less than 1 ng $µl^{-1}$ DNA, nuclease free water in the PCR reaction was adjusted accordingly. The following thermocycling conditions were utilized for the first step PCR: 94°C for 4 min, 94°C for 30 s, 52°C for 45 s, 72°C for 45 s, and a final step of 72°C for 10 min. Triplicates were pooled, purified using the ZR-96 Clean-up kitTM (Zymo Research, Irvine, USA) and 3µl of clean product was used as template in the second PCR (8 cycles) which aimed to insert a sample-specific barcode sequence for subsequent pooling. The 50 µl second PCR reaction contained 32.5 µl nuclease free water, 5 µl 10x DreamTaq Green Buffer, 5 µl 2 mM dNTP mix, 0.25 µl BSA (0.08 µg $µl^{-1}$), 0.25 µl of 1.25U DreamTaq Green DNA Polymerase, 4 µl barcode (0.8µM) and 3 µl of template. After verifying the correct product size by gel electrophoresis, visualized with GelRed Nucleic Acid Stain (Biotium Inc, Fremont, USA) and purifying by ZR-96-Clean-Up Kit (Zymo Research), final products were quantified with Quant-iTTM PicoGreen dsDNA Assay Kit (Thermo Fisher Scientific, Waltham, Massachusetts, USA) and pooled equimolarly (20 x 10-9 copies per sample library).

Supplementary Methods 2

For Shannon diversity index calculations and beta diversity analysis, samples were rarefied to even sampling depth (16S rRNA gene: 1500 reads, ITS1 gene: 1000 reads). Type II ANOVA combined with TukeyHSD test was performed on the Shannon index of the fungal dataset, where the assumption of normality on the residuals was met[1]. For bacterial/archaeal dataset, the Kruskal Wallis rank sum test was used to see differences between the Shannon diversity indices, followed by the Dunn post-hoc test for multiple comparisons of groups from the package “FSA” [2]. Permutational analysis of variance (PERMANOVA) with 9999 permutations from function adonis of the package “vegan” combined with post-hoc pairwiseAdonis was used for testing differences on the Bray-Curtis dissimilarity matrix. For all tests, p-values adjusted by Benjamini-Hochberg corrections are displayed. The analysis of multivariate homogeneity (PERMDIST) was performed using the function betadisper from the package vegan in R with the bias.adjust=T argument which considers sample size differences. [3,4]For comparisons of among-group differences in the distance from observations to their group centroid an ANOVA F-statistic test was used. Significant pairwise comparisons to test which groups were different were performed with a pairwise permutation test for homogeneity of multivariate dispersions with 9999 permutations using the function permutest of the R package “vegan”.

References

1. Kozak, M. & Piepho, H. P. What’s normal anyway? Residual plots are more telling than significance tests when checking ANOVA assumptions. *J. Agron. Crop Sci.* **204**, 86–98 (2018).
2. Ogle, D., Wheeler, P. & Dinno, A. FSA: Fisheries Stock Analysis. *R Packag. version 0.8.31* (2020).
3. Anderson, M. J. Distance-based tests for homogeneity of multivariate dispersions. *Biometrics* **62**, 245–253 (2006).
4. Anderson, M. J., Ellingsen, K. E. & McArdle, B. H. Multivariate dispersion as a measure of beta diversity. *Ecol. Lett.* **9**, 683–693 (2006).
