# Supplementary material for: Both abundant and rare fungi colonizing Fagus sylvatica ectomycorrhizal root-tips shape associated bacterial communities
Source: Commun Biol. 2022 Nov 17;5:1261. doi: 10.1038/s42003-022-04178-y (PMC9672120; doi:10.1038/s42003-022-04178-y)
Supplement: Supplementary file 3 — Description of Additional Supplementary Files [file 42003_2022_4178_MOESM3_ESM.pdf]

Description of Additional Supplementary Files

File name: Supplementary Data 1

Description: Final number of reads per sample used in the analysis.
