# Supplementary material for: Both abundant and rare fungi colonizing Fagus sylvatica ectomycorrhizal root-tips shape associated bacterial communities
Source: Commun Biol. 2022 Nov 17;5:1261. doi: 10.1038/s42003-022-04178-y (PMC9672120; doi:10.1038/s42003-022-04178-y)
Supplement: Supplementary file 5 — Reporting Summary [file 42003_2022_4178_MOESM5_ESM.pdf]

Corresponding author(s): Dagmar Woebken

Last updated by author(s): Oct 11, 2022

Reporting Summary

Nature Portfolio wishes to improve the reproducibility of the work that we publish. This form provides structure for consistency and transparency in reporting. For further information on Nature Portfolio policies, see our Editorial Policies and the Editorial Policy Checklist.

Statistics

For all statistical analyses, confirm that the following items are present in the figure legend, table legend, main text, or Methods section.

n/a	Confirmed	
☐	☒	The exact sample size (*n*) for each experimental group/condition, given as a discrete number and unit of measurement
☐	☒	A statement on whether measurements were taken from distinct samples or whether the same sample was measured repeatedly
☐	☒	The statistical test(s) used AND whether they are one- or two-sided<br>*Only common tests should be described solely by name; describe more complex techniques in the Methods section.*
☒	☐	A description of all covariates tested
☐	☒	A description of any assumptions or corrections, such as tests of normality and adjustment for multiple comparisons
☐	☒	A full description of the statistical parameters including central tendency (e.g. means) or other basic estimates (e.g. regression coefficient) AND variation (e.g. standard deviation) or associated estimates of uncertainty (e.g. confidence intervals)
☐	☒	For null hypothesis testing, the test statistic (e.g. *F*, *t*, *r*) with confidence intervals, effect sizes, degrees of freedom and *P* value noted<br>*Give P values as exact values whenever suitable.*
☒	☐	For Bayesian analysis, information on the choice of priors and Markov chain Monte Carlo settings
☒	☐	For hierarchical and complex designs, identification of the appropriate level for tests and full reporting of outcomes
☒	☐	Estimates of effect sizes (e.g. Cohen's *d*, Pearson's *r*), indicating how they were calculated

Our web collection on statistics for biologists contains articles on many of the points above.

Software and code

Policy information about availability of computer code

Data collection	Sequencing was performed using the Illumina MiSeq platform, v3 chemistry, and 2x300 bp sequencing mode at Microsynth AG (Balgach, Switzerland).
Data analysis	Data analyses and statistics were performed in R (version 3.6.3). Packages “phyloseq” and “vegan” were used for sequence data analyses. Graphs were plotted in “ggplot2”. Package “ggtern” was used for ternary plots and package “UpSetR” was used to create upset plots. Modularity was calculated using the DIRTLPAwb algorithm implemented in the function computModules in the R package "bipartite". "listModuleInformation" in the R package bipartite 2.0 was used to determine the number of modules and identify the bacterial and fungal taxa ascribed to each module. All used R packages are listed and cited in the manuscript. The R codes created to analyze the data will be available upon request.

For manuscripts utilizing custom algorithms or software that are central to the research but not yet described in published literature, software must be made available to editors and reviewers. We strongly encourage code deposition in a community repository (e.g. GitHub). See the Nature Portfolio guidelines for submitting code & software for further information.

Data

Policy information about availability of data

All manuscripts must include a data availability statement. This statement should provide the following information, where applicable:

- Accession codes, unique identifiers, or web links for publicly available datasets
- A description of any restrictions on data availability
- For clinical datasets or third party data, please ensure that the statement adheres to our policy

The sequence data were deposited in the National Center for Biotechnology Information (NCBI) Short Read Archive under study accession no. PRJNA778470 and will be released upon acceptance of the manuscript.

Field-specific reporting

Please select the one below that is the best fit for your research. If you are not sure, read the appropriate sections before making your selection.

☒ Life sciences ☐ Behavioural & social sciences ☐ Ecological, evolutionary & environmental sciences

For a reference copy of the document with all sections, see nature.com/documents/nr-reporting-summary-flat.pdf

Life sciences study design

All studies must disclose on these points even when the disclosure is negative.

Sample size	7 individual trees were used in the study, 8 bulk soil samples, 7 rhisozphere samples. 62 individual mycorrhizal root tips were sampled.
Data exclusions	No data were excluded from the analysis.
Replication	All biological replicates are displayed in the manuscript.
Randomization	Trees used in this study were randomly selected.
Blinding	Blinding was not applicable to this study.

Reporting for specific materials, systems and methods

We require information from authors about some types of materials, experimental systems and methods used in many studies. Here, indicate whether each material, system or method listed is relevant to your study. If you are not sure if a list item applies to your research, read the appropriate section before selecting a response.

Materials & experimental systems

n/a	Involved in the study	
☒	☐	Antibodies
☒	☐	Eukaryotic cell lines
☒	☐	Palaeontology and archaeology
☒	☐	Animals and other organisms
☒	☐	Human research participants
☒	☐	Clinical data
☒	☐	Dual use research of concern

Methods

n/a	Involved in the study	
☒	☐	ChIP-seq
☒	☐	Flow cytometry
☒	☐	MRI-based neuroimaging
